# Patient satisfaction after holmium laser enucleation of the prostate (HoLEP): A prospective cohort study

**DOI:** 10.1371/journal.pone.0182230

**Published:** 2017-08-09

**Authors:** Young Ju Lee, Shin Ah Oh, Sung Han Kim, Seung-June Oh

**Affiliations:** 1 Department of Urology, Seoul National University Hospital, Seoul, South Korea; 2 Department of Urology, Prostate Cancer Center, Research Institute and National Cancer Center, Goyang, South Korea; Taipei Medical University, TAIWAN

## Abstract

**Objective:**

To investigate patient satisfaction after holmium laser enucleation of the prostate (HoLEP) in a prospective study.

**Subjects and methods:**

From May 2012 to December 2014, 397 patients underwent HoLEP by a single surgeon and enrolled in our prospective registry. Baseline data included age, PSA, transrectal ultrasonography, the international prostate symptom score (IPSS), and overactive bladder symptom score (OABSS). Subjective assessment of surgical outcomes was performed at 6 months postoperatively using self-administered questionnaires consisting of ‘satisfaction with treatment question’ (STQ), ‘overall response assessment’ (ORA), and ‘willingness to undergo surgery question’ (WSQ).

**Results:**

A total of 331 patients (mean age 69.6±7.0 years) were included in the analysis. Mean total prostate volume was 69.5 (±42.2) ml. Mean preoperative IPSS score was 18.5 (±7.8). The STQ showed that most patients (91.8%) were satisfied after the surgery. Only 11 (3.3%) patients responded with ‘dissatisfied’, and no patients replied with ‘very dissatisfied’. The WSQ showed that 311 (94.0%) patients were willing to undergo the surgery again if they had to reconsider the surgical decision. The ORA showed that all patients (99.4%) experienced an improvement. When compared with satisfied patients, neutral/dissatisfied patients had lower IPSS quality of life scores (2.7 vs. 0.9, p<0.001), higher IPSS voiding symptom scores (7.0 vs. 1.4, p<0.001), and more frequent episodes of urgency urinary incontinence in OABSS (1.0 vs. 0.3, p = 0.017) at 6 months postoperatively.

**Conclusions:**

The overall level of satisfaction after HoLEP was high. The most common reason for dissatisfaction was the occurrence of urgency urinary incontinence after the surgery.

## Introduction

Benign prostatic hyperplasia (BPH) is a major cause of lower urinary tract symptoms (LUTS) in men. It is a common urogenital problem affecting men over the age of 50 years. Transurethral resection of the prostate (TURP) has been regarded as a gold standard for the treatment of BPH. Due to advancements in technology, minimally invasive surgery has become popular [1, 2], and laser-based prostatectomy is expected to become an alternative to monopolar TURP or open prostatectomy.

Holmium laser resection of the prostate combined with mechanical morcellation was first described by Gilling et al. in 1998 [3]. Through improvements to the holmium laser and morcellator technologies [4], significantly better postoperative results were obtained with holmium laser enucleation of the prostate (HoLEP) than with TURP [5]. Although HoLEP has a steep learning curve [6], it is the only laser treatment that has considerable supporting level 1 evidence and has been recommended by the American Urological Association and European Association of Urology. The efficacy of HoLEP is comparable with that of TURP for smaller prostates and comparable with that of open prostatectomy for larger prostates with a lower risk of complications [7]. HoLEP is suggested to be a new gold standard for BPH treatment [8].

Improvements in objective perioperative parameters after HoLEP, such as the maximal flow rate, the international prostate symptom score (IPSS), and the quality of life (QoL) scores have been previously shown [9]. However, unlike objective outcomes, only a few studies have addressed subjective outcomes such as patient satisfaction [10]. Global assessments of treatment benefit, satisfaction with the treatment and willingness to continue to use a treatment are determinants of treatment effectiveness [11].

Therefore, we investigated patient satisfaction after HoLEP with questionnaires after enrolling patients in a prospectively collected database registry.

## Subjects and methods

This study was approved by the Institutional Review Board at Seoul National University Hospital (IRB No. 0810-027-260).

### Subjects

A total of 397 patients who underwent HoLEP at our institution from May 2012 to December 2014 were enrolled in a database registry after providing written consents according to the approval of Institutional Review Board. The Seoul National University Hospital Benign Prostatic Hyperplasia Database Registry is a prospectively collected database of BPH patients aged 50 years or more. Patients who had not provided consent or those with prostate cancer were excluded. Digital rectal examination, 3-day voiding diary records, IPSS, overactive bladder symptom score (OABSS), urinalysis, prostate-specific antigen (PSA), uroflowmetry, transrectal ultrasound of the prostate, urodynamic study, and cystourethroscopy were performed as a baseline study. Surgery data including surgery duration and enucleated tissue weight were recorded. Data for IPSS, OABSS, 3-day voiding diary records, urinalysis, and uroflowmetry were obtained at follow-up visits at 2 weeks, 3 months, and 6 months postoperatively. At 6 months postoperatively, the PSA levels were determined and patient satisfaction questionnaires were administered. The self-administered questionnaires composed of a ‘satisfaction with treatment question’ (STQ), an ‘overall response assessment’ (ORA), and a ‘willingness to undergo surgery question’ (WSQ) (S1 Table). Questionnaires were used after linguistic validation. Linguistic validation was performed before the beginning of this research. A total of 5 patients with BPH participated in the debriefing process. It was concluded that there was no difficulty in understanding and answering to the questionnaire. All data were collected independently by a single study coordinator at 2 weeks, 3 months, and 6 months postoperatively. The physician was blinded to the STQ, ORA, and WSQ responses.

The indications for HoLEP were as follows: Absolute indications were the presence of bladder stones, gross haematuria due to BPH, recurrent urinary tract infections, and recurrent acute urinary retention. Relative indications were severe voiding difficulty due to significant bladder outlet obstruction determined by the urodynamic study during the pressure-flow study or persistent LUTS refractory to other therapies. Each patient evaluation and the surgical decision were in accordance with AUA and EAU guidelines [12, 13]. Bladder outlet obstruction was diagnosed based on the result of pressure-flow study. Patients with obvious neurogenic bladder, severe urethral stricture, or genitourinary malignancy were excluded. However, patients with previous history of minimal or no neuropathies such as lacunar infarct, transient ischemic attack, Parkinsonism without significant morbidity, and diabetes mellitus without peripheral neuropathy were included.

The surgical procedures were performed by a single surgeon in the same way as previously described [14]. Briefly, HoLEP was performed with the patient in a dorsal lithotomy position and under spinal or general anaesthesia. The Ho:YAG laser (VersaPulse, Lumenis, Yokneam, Israel) was set to 80 W (2 J, 40 Hz). After the initial incisions in the 5- and 7-o’clock directions, a transverse incision was made just proximal to the verumontanum. After the removal of the median lobe, the lateral lobes were removed, completing the enucleation process. After careful haemostasis, morcellation was performed by using a 26-Fr nephroscope and a tissue morcellator (Versacut^TM^, Lumenis). A 22-Fr 3-way urethral catheter was placed with continuous normal saline irrigation (≥60 gtt) and removed on postoperative day 1 or 2. Patients were usually discharged at postoperative day 1 unless there was significant hematuria or unless the surgery was performed late at night. Follow-up visits were made at 2 weeks, 3 months, and 6 months postoperatively on an outpatient basis. Thereafter, the patients were instructed to discontinue follow-up visits unless a major medical event occurred.

### Statistical analysis

The individual variables are expressed as mean ± SD. Paired t-tests were used to compare the postoperative changes in values. To compare between the groups of dissatisfied and satisfied patients, independent Student’s t-tests were used for continuous variables and chi-square tests were used for discrete variables. Univariate and multivariate logistic regression analyses were used for the comparison of variables according to patient satisfaction. IBM SPSS version 22.0 (SPSS Inc., Chicago, IL) was used for the statistical analysis. P values less than 0.05 were considered significant.

## Results

Among the 397 patients, 331 (83.4%) were followed up at 6 months postoperatively and were included in the analysis. The mean age was 69.6 ± 7.0 years (range 53–87). Mean prostate volume was 69.5 ± 42.2 ml. The socio-economic characteristics of the patients are shown in Table 1. Patient characteristics in relation to clinical parameters are presented in Table 2. Q_max_ increased significantly after the surgery, while the PSA levels, the postvoid residual volumes, and the IPSS scores decreased significantly. QoL improved significantly after the surgery.

**Table 1:** Characteristics of patients.

Demographics		No. of patients (%)
Previous medical history		
	Hypertension	141 (42.6%)
	Diabetes	66 (19.9%)
	Neurologic disease	46 (13.9%)
	Cerebrovascular disease	35 (10.6%)
Level of education		
	Elementary school	32 (9.7%)
	Middle school	38 (11.5%)
	High school	95 (28.7%)
	College	160 (48.3%)
Occupation		
	Unemployed	117 (35.3%)
	Retired	62 (18.7%)
	Bussiness/professional	53 (16.0%)
	Sales/service/white-collar	49 (14.8%)
	Farming/fisheries/forestry	30 (9.1%)
	Technical/manufacturing/blue-collar	18 (5.4%)
Household income (won/month)		
	Less than a million	58 (17.5%)
	1–2 million	72 (21.8%)
	2–4 million	93 (28.1%)
	4–6 million	51 (15.4%)
	Over 6 million	42 (12.7%)

**Table 2:** Perioperative change of clinical parameters after the surgery.

Variable	Preoperative	3 months after surgery	6 months after surgery	P value*
PSA (ng/dL)	3.94±4.17	N/A	1.02±1.23	<0.001
Qmax (ml/sec)	9.5±4.7	21.6±10.7	22.4±10.5	<0.001
PVR (ml)	65.1±89.4	21.3±36.6	21.5±73.4	<0.001
Total IPSS	18.5±7.8	7.2±5.8	5.1±5.0	<0.001
IPSS-Storage	7.5±3.5	4.6±2.9	3.3±2.6	<0.001
IPSS-Voiding	11.0±5.4	2.6±3.6	1.9±3.1	<0.001
IPSS 8 (QoL)	4.0±1.3	1.5±1.5	1.1±1.2	<0.001
OABSS	6.2±3.5	3.8±3.0	2.8±2.6	<0.001

TPV, Total prostatic volume; PVR, Postvoid residual volume; IPSS, International Prostate Symptom Score; OABSS, Overactive Bladder Symptom Score; N/A, not available; *paired t-test for preoperative and postoperative 6 months

**Fig 1:**
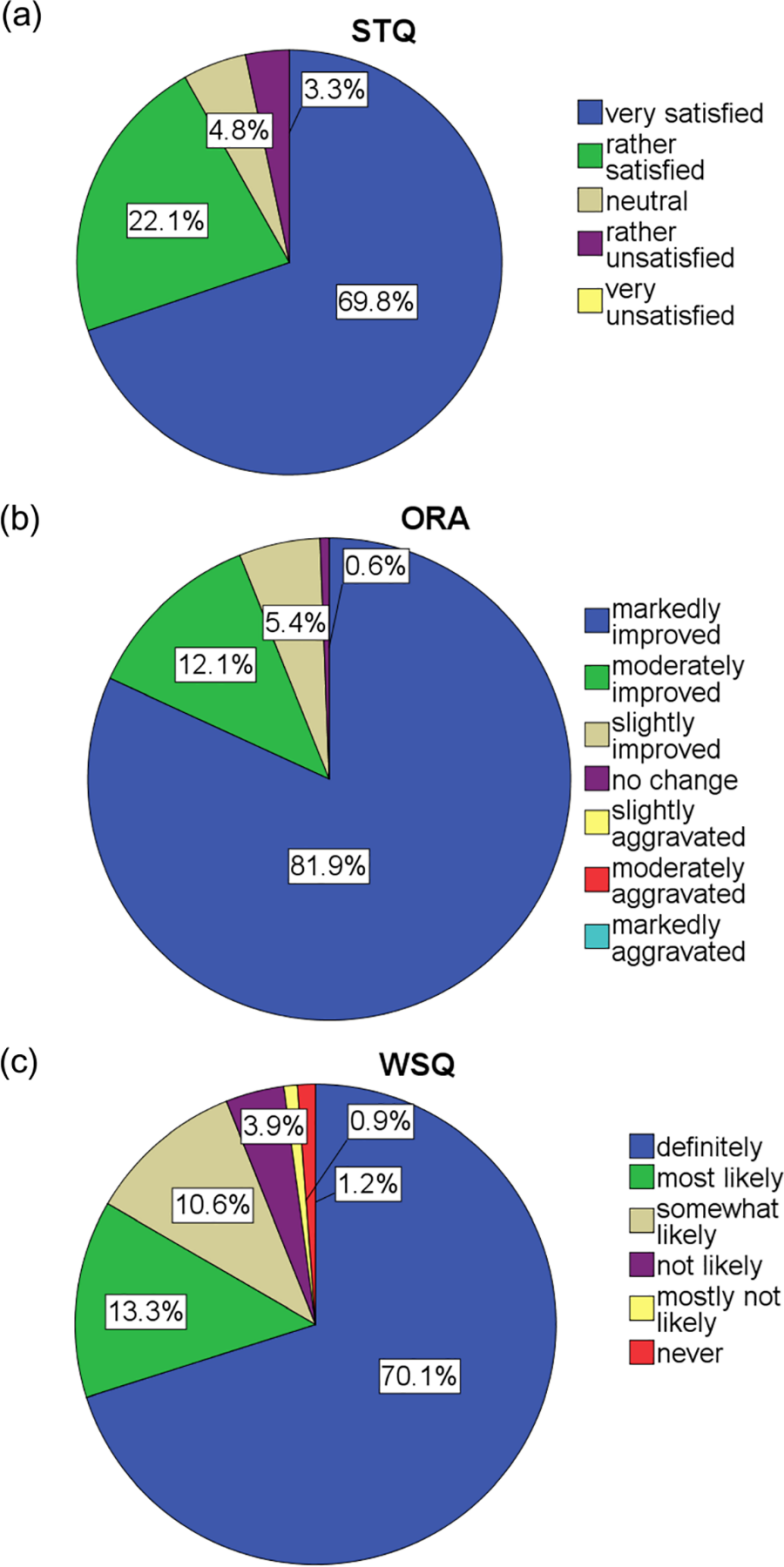
The results of the self-administered questionnaires. (a) Satisfaction with treatment question (STQ), (b) overall response assessment (ORA), (c) willingness to undergo surgery question (WSQ)

The results of the self-administered questionnaires are shown in Fig 1. Among the 331 patients, 304 (91.8%) patients were satisfied. Only 11 (3.3%) patients responded that they were ‘dissatisfied’ and no patients responded with ‘very dissatisfied’. The reasons for dissatisfaction were as follows: postoperative transient incontinence (n = 3) including 1 stress urinary incontinence, increased daytime frequency (n = 6), feeling of incomplete emptying (n = 1), and slow stream (n = 1). Responses of ‘neutral’ were accompanied with the following reasons: postoperative incontinence (n = 3) including 1 transient incontinence and 1 minimal wetting, 1 urgency incontinence which was improved at 6 months, increased daytime frequency (n = 7), feeling of incomplete emptying (n = 1), slow stream (n = 3), and retrograde ejaculation (n = 1), occasional urgency (n = 1). Patients who could not void spontaneously and required clean intermittent catheterisation (CIC) preoperatively (n = 8) or those with a previous history of BPH surgery (n = 16) were all satisfied after HoLEP. History of diabetes, cerebrovascular disease, cerebral or spinal disease, and Parkinsonism were not associated with patient satisfaction (p > 0.05).

For the ORA, only 2 (0.6%) patients reported no change, of which one was dissatisfied due to persistent nocturia and the other patient responded with ‘neutral’ because of postoperative transient urinary incontinence. Most patients reported an improvement after the surgery. No one reported symptom aggravation after the surgery. For the WSQ, 311 (94.0%) patients expressed willingness to undergo the surgery, whereas 20 (6.0%) patients did not want the surgery. The levels of satisfaction for the 20 unwilling patients were as follows: very satisfied (n = 5), satisfied (n = 5), neutral (n = 5), and dissatisfied (n = 5).

Table 3 shows the patient characteristics according to patient satisfaction levels. Dissatisfied patients tended to have more severe voiding symptoms postoperatively. The IPSS and OABSS scores at 6 months postoperatively were significantly higher in the dissatisfied group than in the satisfied group, although the preoperative values did not differ between the 2 groups. The improvement in maximal flow rate, IPSS voiding and QoL scores, and nocturia was significantly higher in the satisfied group than in the dissatisfied group. Dissatisfied patients were more likely to have worse ORA (OR 11.92, 95% CI 6.10–23.28, p < 0.001). Dissatisfied patients tended to show reduced willingness to undergo surgery (OR 3.13, 95% CI 2.20–4.47, p < 0.001). Multivariate logistic regression analysis for patient satisfaction showed neutral/dissatisfied patients tended to have a higher IPSS voiding score at postoperative 6 months (OR 1.37, 95% CI 1.19–1.58; p < 0.001) and have no willingness to undergo the surgery again (OR 10.64, 95% CI 3.10–36.40; p < 0.001) when adjusted with age, history of neurologic disease, total prostatic volume and IPSS storage score at postoperative 6 months (Table 4).

**Table 3:** Clinical characteristics of patients according to patient satisfaction.

Variable		Satisfied (n = 304)	Neutral/dissatisfied (n = 27)	P-value
Age (years)		69.5±7.1	70.7±6.1	0.428
BMI (kg/m^2^)		24.1±2.6	24.0±3.3	0.894
TPV (ml)		70.6±42.6	56.9±35.8	0.113
Preoperative				
	Qmax (ml/sec)	9.5±4.6	8.4±3.5	0.282
	PVR (ml)	62.8±86.8	73.1±94.0	0.557
	IPSS-total	18.1±7.7	22.6±7.8	0.428
	IPSS-voiding	10.8±5.4	13.3±5.2	0.894
	IPSS-storage	7.3±3.4	9.4±3.7	0.113
	OABSS-sum	6.2±3.4	7.1±3.7	0.282
Postoperative 3 months				
	Qmax (ml/sec)	22.1±10.7	15.1±8.8	0.003
	PVR (ml)	20.4±35.1	33.6±52.0	0.103
	IPSS-total	6.8±5.6	11.7±5.9	<0.001
	IPSS-voiding	2.4±3.5	5.5±4.4	0.002
	IPSS-storage	4.5±2.9	6.2±3.1	0.004
	OABSS-sum	3.7±3.0	5.3±3.7	0.012
Postoperative 6 months				
	Qmax (ml/sec)	22.6±10.5	15.9±7.7	**0.003**
	PVR (ml)	19.6±68.7	52.7±110.2	0.152
	IPSS-total	4.4±4.1	13.0±6.9	**<0.001**
	IPSS-voiding	1.4±2.4	7.0±5.3	**<0.001**
	IPSS-storage	3.0±2.4	6.0±3.4	**<0.001**
	OABSS-sum	2.5±2.4	5.2±3.2	**<0.001**
Changes 6 months after operation				
	Δ IPSS-total	-13.7±7.9	-9.6±7.5	**0.011**
	Δ IPSS-voiding	-9.4±5.6	-6.3±5.6	**0.006**
	Δ IPSS-storage	-4.3±3.4	-3.3±3.3	0.156
	Δ IPSS-QoL	-3.1±1.6	-1.7±1.5	**<0.001**
	Δ OABSS sum	-3.6±3.5	-1.9±3.6	**0.018**
	Δ OABSS #1 (Frequency)	-0.4±0.7	-0.1±0.7	0.128
	Δ OABSS #2 (Nocturia)	-0.8±0.9	-0.4±0.9	**0.033**
	Δ OABSS #3 (Urgency)	-1.7±1.8	-1.0±2.0	0.086
	Δ OABSS #4 (UUI)	-0.8±1.5	-0.4±1.9	0.146
	Δ Qmax (ml/sec)	13.4±10.4	7.1±8.5	**0.009**
	Δ PVR (ml)	-45.7±109.6	-23.1±58.0	0.312

BMI, Body Mass Index; TPV, Total prostatic volume; PVR, Postvoid residual volume; Δ, changes before and 6 months after HoLEP

**Table 4:** The result of multivariate logistic regression analysis for patient satisfaction. Neutral/dissatisfied patients were compared with satisfied patients.

Variables	OR (95% CI)	p-value
Age	0.98 (0.90–1.07)	0.610
Total prostate volume (ml)	0.98 (0.96–1.01)	0.111
History of neurologic disease	2.16 (0.64–7.32)	0.215
IPSS voiding score at 6 months	1.37 (1.19–1.58)	<0.001
IPSS storage score at 6 months	1.18 (0.98–1.42)	0.086
Willingness to undergo the surgery again	0.09 (0.03–0.32)	<0.001

## Discussion

Patient-reported outcomes are important in measuring surgical outcomes, especially for benign diseases. Traditional surgical outcomes of morbidity and mortality are important, but not sufficient to report the outcomes for minor surgeries for benign diseases. The outcomes of transurethral prostatectomy for BPH were measured previously by changes in maximal flow rate, postvoid residual volumes, and changes in symptom scores using IPSS. Patient-reported outcomes such as quality of life and satisfaction are also important issues after transurethral prostatectomy.

Treatment benefit, satisfaction, and willingness to continue a treatment are all attributes for a successful treatment [11]. The Benefit, Satisfaction, and Willingness (BSW) questionnaire is a valid outcome measurement previously used in clinical trials for overactive bladder and anti-incontinence surgeries [15, 16]. In the previous article, evaluating patient satisfaction after HoLEP used a single questionnaire to measure the satisfaction level [10]. In our study, we developed a 3-item questionnaire after debriefing to evaluate the treatment efficacy and preference after HoLEP. STQ, ORA and WSQ assess subjective satisfaction, symptomatic overall improvement, and willingness to undergo the surgery again after the surgery and the recovery period. Those three questionnaires evaluate patient satisfaction after HoLEP in a multifactorial way similar to the BSW. The term ‘satisfaction’ is highly individualized and includes many variables regarding treatment such as cost, risks, benefits, expectations, and also includes procedural factors, such as any inconvenience, treatment-related experiences, objective or subjective outcomes, and a combination of these factors [17]. ORA evaluates subjective improvement and WSQ inquires including the treatment-related experiences and inconveniences.

It is well known that neither prostate volume nor the presence of bladder outlet obstruction is associated with subjective LUTS [18]. Although absolute indications may be present, patient inconvenience is still a major factor in the consideration of surgery [19]. Our results revealed the gap between symptom improvement and satisfaction, showing the subjectivity of patient satisfaction. Although most patients reported improvements after the surgery, a few of them were dissatisfied due to persistent storage symptoms or unmet expectations. A patient-centred individualised approach is necessary to select the appropriate surgical candidate and to manage patient expectations in a realistic way. Additionally, it is noteworthy that patients who had previous BPH surgery or preoperative CIC were all satisfied after the surgery, and this reflects the importance of patient expectations.

Previously, TURP was the gold standard for the minimally invasive therapy of BPH. Meyhoff et al. compared the satisfaction rate of patients after they underwent TURP and transvesical prostatectomy and showed that more than 90% of the patients were satisfied after 5 years [20]. In their study, the satisfaction rate at 6 months after undergoing TURP was 85%, and urge incontinence was the main reason for the dissatisfaction [21]. Ala-Opas et al. also reported a 92% satisfaction rate for TURP after a mean of 6.5 years, and incontinence and urgency were the reasons for dissatisfaction [22]. Mishiriki et al. reported that 8.4%, 20.8%, and 13.3% of patients were dissatisfied after TURP at 6 months, 6 years, and 12 years, respectively [23]. Malek et al. reported that 90% of the patients were very satisfied with potassium titanyl-phosphate photoselective vaporisation of the prostate [24], whereas Ku et al. reported that 37 of the 97 patients were dissatisfied at 12 months postoperatively, and the reason for dissatisfaction was weak detrusor contractility [25].

Previous studies in HoLEP demonstrated an increase in maximal flow rate and a decrease in postvoid residual volume after HoLEP [5, 26, 27], and QoL improved significantly [28]. These were consistent with our results. To evaluate patient satisfaction after HoLEP, Gilling et al. conducted a phone survey of participants of HoLEP arms of prospective trials at 6 years postoperatively [10]; among the 71 participants, 38 provided responses and 92% of them were satisfied with the outcomes. In comparison with this previous study, our study is more robust due to its prospective design, relatively larger number of patients, and lower dropout rate. In addition, ORA and WSQ responses were evaluated.

Postoperative urinary incontinence is one of the concerns for surgeons performing HoLEP [29]. Transient urinary incontinence, defined as any type of urine leakage, mostly disappear within 3 months postoperatively [30]. In our study, some of dissatisfied or neutral patients reported that urinary incontinence was the reason affecting their satisfaction levels. Because no learning curve issue existed during the study period, surgeon’s experience might not be the factor affecting the satisfaction.

In our study, a few patients had a history of stroke, Parkinsonism, cerebrospinal surgeries, and diabetes. However, the conditions were not severe, and the patients were ambulatory and capable of understanding our explanations and expressing their willingness to participate in the study. Those patients were enrolled in this study because evidence of bladder outlet obstruction was a major indication for HoLEP. Our results showed that the presence of these diseases was not associated with dissatisfaction.

There could be a few reasons for the high satisfaction rate. The provision of a patient-centred individualised approach was a key factor; we followed a protocol before the surgery that outlined a systematic approach aimed at minimising unmet expectations and maximising patient satisfaction. Comprehensive explanations were provided at the outpatient clinic after the decision to perform surgery was made. The preoperative counselling included information such as the benefit of HoLEP, previous surgical results, complication rates, surgical process, and the cost. It is very important to have appropriate surgical indications and select the right surgical candidates. In addition to the traditional absolute surgical indications, we believe that the presence of bladder outlet obstruction determined by urodynamic studies is a key surgical indication.

It is known that HoLEP removes adenomas in the same anatomical way as open prostatectomy does. All patients reported symptomatic improvements. However, postoperative irritative voiding symptoms, such as dysuria, are more prevalent after HoLEP than after TURP [5, 31]. In our study, urinary incontinence was the second most common reason for dissatisfaction. Previous studies have also reported similar findings; although transient urinary incontinence might be more common after HoLEP, there were no significant differences between HoLEP and TURP in terms of urinary incontinence after the first year [32, 33]. Because we studied patient satisfaction at 6 months postoperatively, these patients might have experienced an improvement in symptoms after then.

Our study has a few limitations. This study was performed in a non-comparative design with a relatively short term follow-up. Questionnaires were administered at 6 months postoperatively, which is a relatively short follow-up period when compared previous long-term follow-up studies. However, considering the durable surgical effect and improvements after HoLEP [10, 26, 28], a 6-month period may not be inappropriate for the evaluation. Patients responded to the questionnaires after completing their last visit at postoperative 6 months. In addition, all the responses from the patients were blinded to the surgeon. We believe these have minimized potential bias. Future studies with a larger number of patients and longer follow-up periods would be useful.

In conclusion, the satisfaction rate after HoLEP was found to be high, and most patients experienced symptomatic improvement. Appropriate surgical decisions as well as patient-centred treatment are important to achieve this result.

## Supporting information

S1 Table Questionnaires of satisfaction with treatment question, overall response assessment and willingness to undergo surgery question. (DOCX)
