# Supplementary material for: Patient satisfaction after holmium laser enucleation of the prostate (HoLEP): A prospective cohort study
Source: PLoS One. 2017 Aug 9;12(8):e0182230. doi: 10.1371/journal.pone.0182230 (PMC5549990; doi:10.1371/journal.pone.0182230)
Supplement: S1 Table — (DOCX) [file pone.0182230.s001.docx]

S 1 Table. Questionnaires of Satisfaction with Treatment Question, Overall Response Assessment and Willingness to undergo Surgery Question.

| Satisfaction with Treatment Question | Overall Response Assessment | Willingness to undergo Surgery Question |
| --- | --- | --- |
| a. Very satisfied  b. Satisfied  c. Neutral  d. dissatisfied  e. Very dissatisfied | a. Markedly improved  b. Moderately improved  c. Slightly improved  d. No change  e. Slightly worse  f. Moderately worse  g. Markedly worse | a. Definitely  b. Most likely  c. Somewhat likely  d. Not likely  e. Mostly not likely  f. Never |
